# Temozolomide and Other Alkylating Agents in Glioblastoma Therapy

**DOI:** 10.3390/biomedicines7030069

**Published:** 2019-09-09

**Authors:** Hannah Strobel, Tim Baisch, Rahel Fitzel, Katharina Schilberg, Markus D. Siegelin, Georg Karpel-Massler, Klaus-Michael Debatin, Mike-Andrew Westhoff

**Affiliations:** 1 Department of Pediatrics and Adolescent Medicine, University Medical Center Ulm, D-89075 Ulm, Germany (H.S.) (T.B.) (R.F.) (K.-M.D.); 2 Faculty of Medicine, Ulm University, D-89081 Ulm, Germany; 3 Department of Pathology and Cell Biology, Columbia University Medical Center, New York, NY 10032, USA; 4 Department of Neurosurgery, University Medical Center Ulm, D-89081 Ulm, Germany

**Keywords:** temozolomide (TMZ), triazene compounds, alkylating agents, brain tumor, glioblastoma

## Abstract

The alkylating agent temozolomide (TMZ) together with maximal safe bulk resection and focal radiotherapy comprises the standard treatment for glioblastoma (GB), a particularly aggressive and lethal primary brain tumor. GB affects 3.2 in 100,000 people who have an average survival time of around 14 months after presentation. Several key aspects make GB a difficult to treat disease, primarily including the high resistance of tumor cells to cell death-inducing substances or radiation and the combination of the highly invasive nature of the malignancy, i.e., treatment must affect the whole brain, and the protection from drugs of the tumor bulk—or at least of the invading cells—by the blood brain barrier (BBB). TMZ crosses the BBB, but—unlike classic chemotherapeutics—does not induce DNA damage or misalignment of segregating chromosomes directly. It has been described as a DNA alkylating agent, which leads to base mismatches that initiate futile DNA repair cycles; eventually, DNA strand breaks, which in turn induces cell death. However, while much is assumed about the function of TMZ and its mode of action, primary data are actually scarce and often contradictory. To improve GB treatment further, we need to fully understand what TMZ does to the tumor cells and their microenvironment. This is of particular importance, as novel therapeutic approaches are almost always clinically assessed in the presence of standard treatment, i.e., in the presence of TMZ. Therefore, potential pharmacological interactions between TMZ and novel drugs might occur with unforeseeable consequences.

## 1. Introduction

Temozolomide (TMZ), also known by its tradenames Temodal^®^ and Temodar^®^, is an alkylating agent belonging to the group of triazene compounds. Together with maximal safe tumor debulking and focal radiotherapy, this drug is an essential part of the current standard treatment of glioblastoma (GB), a particular aggressive type of primary brain tumor, which essentially must be considered an incurable disease [1]. In particular, the highly invasive growth pattern of GB makes it impossible to completely remove the tumor by surgical resection without impairing the patient’s brain function, which ultimately results in tumor recurrence and death of the patient [2].

The addition of TMZ to the standard treatment protocol was hailed as a major breakthrough in GB therapy, although it only prolonged the median overall survival of GB patients to 14.6 months compared to 12.1 months with radiation therapy alone [3,4]. Despite this improvement in therapy, patients’ prognosis remains dismal with a five-year overall survival below 10% [3,5].

Novel treatment approaches, which are clearly urgently needed, are currently being investigated in a preclinical setting as well as in clinical trials; for example, the National Institutes of Health database at clinicaltrials.gov lists 477 trials as active or at various stages of the recruitment procedure. However, every new approach is evaluated in the presence of the current standard of care; for obvious reasons, oncological trials are not performed with a placebo group. Therefore, it is essential to understand the mode of action with regard to TMZ to ascertain potential synergistic or antagonistic interactions between this treatment and novel approaches.

In this review, we collated the available information on TMZ and compared primary data to claims made frequently in the literature. This allowed us to re-evaluate the role of TMZ in GB therapy and identify potential pitfalls in future treatment evaluations.

## 2. Alkylating Agents—Their Chemistry and Biological Uses

As suggested by their names, alkylating agents contain reactive alkyl groups that are composed of carbon and hydrogen atoms defined by the general formula C_n_H_2n+1_ [6]. Alkylating agents most commonly transfer their electrophilic alkyl groups to ring nitrogen and extracyclic oxygen atoms of the DNA bases, as those are the most nucleophilic atoms within the DNA [7].

In general, alkylating agents can be classified according to the number of reactive sites, their chemical reactivity, and the type of alkyl group added. Monofunctional agents contain only one active group, which is why only one site within the DNA can be modified. In contrast, bifunctional agents carry two reactive groups by which interstrand DNA crosslinks can be formed as well [8,9]. Interstrand crosslinks, for instance, prevent uncoiling of DNA during cell division; therefore, not surprisingly, bifunctional agents are highly cytotoxic [6,10].

Traditionally, alkylating agents are further classified according to their chemical reactivity. In general, two reaction kinetics are distinguished: S_N_1 versus S_N_2 kinetics. In S_N_1-type reactions, a carbocation intermediate is formed first, which is subsequently attacked by the nucleophilic group of the DNA. This type of reaction is unimolecular, meaning that the rate of the reaction only depends on the concentration of the reactive intermediate. S_N_2-type reactions, on the other hand, occur in only one step. Consequently, the rate of the reaction, also termed a bimolecular reaction, depends on the concentrations of both reactants [10]. The S_N_1-and the S_N_2-reaction mechanisms have been used to explain differences in the proportions of adducts formed at oxygen and nitrogen atoms in DNA [8,9,11]. S_N_2-type reactions tend to predominantly occur at ring nitrogen atoms such as N7 in guanine, which possesses the greatest negative electrostatic potential [8,12]. S_N_1-type reactions, on the other hand, tend to proceed with nitrogen and oxygen atoms [8]. However, the predictive potential of this theory is often limited, especially when considering complex alkylating agents [11,13]. Therefore, alternative approaches are being investigated in order to predict reaction products [14,15]. 

Another classification aspect for alkylating agents is the type of alkyl group that can be transferred. The alkyl groups range from simple methyl groups (CH_3_) or chloroethyl groups (C_2_H_4_-Cl) to more complex alkyl groups, as is the case for mitomycin C, for instance [9,16].

Owing to cytostatic and cytotoxic effects induced by alkylation damage, alkylating agents are nowadays commonly used as chemotherapeutic drugs in cancer therapy. A class of alkylating agents with similar properties and of particular clinical interest are the triazene compounds, for example, the antimelanoma drug dacarbazine (DTIC), mitozolomide, and TMZ [17,18]. 

## 3. Triazene Compounds and Their Mode of Action

Triazenes are defined by the presence of a triazenyl group (RN = N-NR′R″) as an active moiety [18]. The first suggestion of their anticancer activity arose in 1955, when Clarke and colleagues showed that 3,3-dimethyl-phenyltriazenes could inhibit sarcoma growth in mice [19].

**Figure 1:**
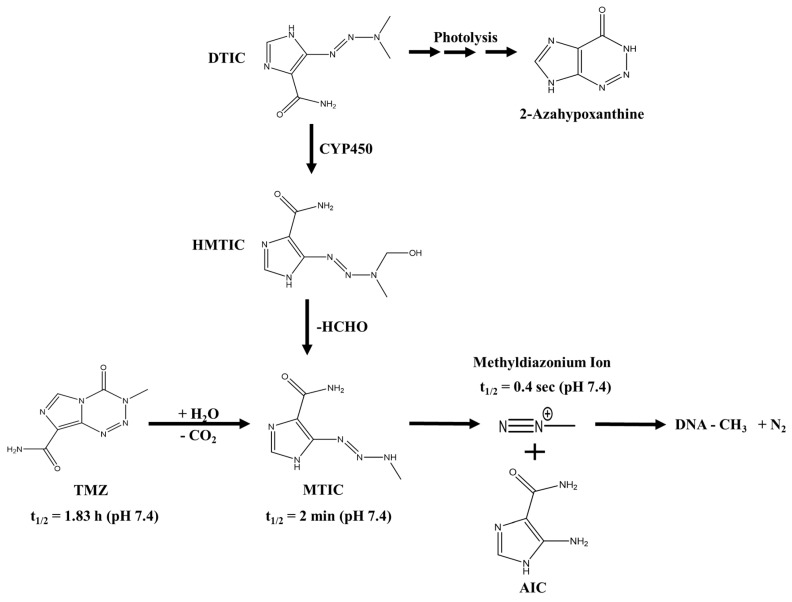
Prodrug activation of TMZ and DTIC. DTIC requires oxidative N-demethylation via CYP450 enzymes, giving rise to HMTIC. HMTIC then eliminates formaldehyde and forms MTIC. TMZ, in contrast, is spontaneously hydrolyzed to MTIC at neutral or alkaline pH. Thereafter, DTIC and TMZ share the same pathway. MTIC further fragments to AIC and the methyldiazonium ion, which in turn reacts with nucleophilic sites, for example, in the DNA. In addition, DTIC can be activated by photolysis, yielding 2-azahypoxanthine. Abbreviations: AIC, 5-aminoimidazole-4-carboxamide; CYP450, cytochrome P450; DNA, deoxynucleic acid; DTIC, dacarbazine; HMTIC, 5,3-hydroxy,ethyl-3-methyl-triazene-imidazole-carboxamide; MTIC, 5-(3-methyltriazen-1-yl)-imidazole-4-carboxamide; TMZ, temozolomide. Based on [18,32].

However, the big breakthrough in this area happened in 1959, when researchers of the University of Alabama synthesized DTIC, an imidazole triazene where the triazene is fused to an imidazole ring system. As it showed potent activity against a range of tumors in rodent models, DTIC was launched into clinical practice in the 1970s for the treatment of metastatic melanoma and can at best be described as only a modest performer in clinical practice [17,18]. It has been suggested that these rather disappointing results in patients are, at least in part, attributable to the lower activity of hepatic enzymes such as the cytochrome P450 (CYP450) family in humans compared to rodents. This in turn leads to a reduced metabolic conversion of DTIC to its active metabolite 5-(3-methyl-1-triazeno)imizadole-4-carboxamide (MTIC) [20,21,22]. The similarities and the differences between DTIC and TMZ activation are summarized in Figure 1.

In the late 1970s, researchers at the Aston University synthesized new imidazotetrazinones (organic heterobicyclic compounds containing ortho-fused imidazole and tetrazine rings) and screened them against mouse tumors [17,21]. The lead compound mitozolomide showed great efficacy in many rodent models but failed in clinical practice due to its unfavorable toxicity profile in humans, which was mainly attributed to its DNA cross-linking ability [23,24]. In phase I studies, dose-limiting toxicity in form of thrombocytopenia was identified [24]. Phase II studies showed that mitozolomide caused unpredictable myelotoxicity at the recommended dose of 150 mg/m^2^ and even at the reduced dose of 90 mg/m^2^ [25,26]. Therefore, when selecting the “second-generation” of imidazotetrazinones, TMZ, a monofunctional S_N_1-type methylating agent, less toxic but also less potent than mitozolomide, was chosen out of the pool of analogues [17,21]. TMZ was first used to treat glioma patients in a phase I trial at the Charing Cross Hospital in 1987. The initial results were rather disappointing but were attributed to the schedule dependency of TMZ. After optimization of the dose-schedule, TMZ was shown to benefit glioma and metastatic melanoma patients [17]. Finally, in 1999, TMZ was approved for recurrent GB and anaplastic astrocytoma by the Food and Drug Administration (FDA) and the European Medicines Agency. Following clinical trials performed by Stupp and colleagues in 2005, TMZ was also approved for first-line therapy of newly diagnosed GB [3]. Additionally, TMZ is used “off-label” in metastatic melanoma patients [27].

Although TMZ has now been used in the clinic for more than a decade to treat GB, the molecular mechanisms underlying TMZ-based action are still not completely understood and controversially discussed in the literature [28,29]. TMZ is a prodrug that is, in contrast to many other chemotherapeutic substances, able to cross the blood brain barrier (BBB) and reaches the tumor site in therapeutically relevant concentrations [30,31]. Unlike other triazene compounds, TMZ does not require metabolic activation and is spontaneously converted to the active compound at blood pH [32,33,34]. In an intermediate step, TMZ is spontaneously converted to MTIC, which is also present after metabolic activation of DTIC, but here, the conversion is dependent on enzymes. This might, at least in part, explain the poor performance of DTIC in the clinics. Rodent CYP450 enzymes are expressed in different tissues and possess different catalytical activity and specificity compared to the human enzymes [35]. In line with this finding, mice and rats seem to have a greater metabolism of DTIC to MTIC, causing greater therapeutic effects when compared to the ones observed in men [20]. Furthermore, as DTIC requires metabolic activation, it is subject to inter-patient variability in terms of hepatic metabolism, which can be heavily influenced in cancer patients due to the intake of several drugs at the same time (e.g., anticonvulsants for seizure management and corticosteroids for oedema management) [36,37,38]. The active compound is the electrophilic methyldiazonium cation, which is able to methylate the DNA mostly at guanine residues [30,32,34]. DNA methylation can occur at the N7 (60–80%) or the O^6^ (5%) position of guanine as well as the N3 (10–20%) position of adenine [33,39]. The most frequent DNA N-methylations are effectively repaired by the base excision repair (BER) pathway, which counteracts TMZ-induced DNA damage. Thus, a functional BER, which is only rarely inactivated in GB tumors, contributes to TMZ resistance and is associated with a worse prognosis in GB patients [39]. Probably counterintuitively, antitumoral activity of TMZ requires a functional DNA mismatch repair (MMR) of the tumor cell and is mediated by the lower frequent O^6^-methylguanine lesions [33,40]. O^6^-methylguanine is mispairing with thymine, which is recognized by the MMR machinery [39,40]. The mispairing thymine is excised and replaced with another thymine upon repair synthesis thus leading to futile, energy-consuming cycles of DNA repair [41,42]. The methylated guanine, which cannot be repaired by MMR, persists on the opposite strand, leading to a replication fork arrest and presumably to DNA double strand breaks and eventual apoptosis [39,40,43]. The work of Hirose and colleagues hints at a mainly cytostatic effect of TMZ, because cells accumulate in G2/M phase upon TMZ treatment, thereby contrasting the model of TMZ-mediated cytotoxicity [41,42].

However, O^6^-methylguanine adducts can be directly repaired by the suicide enzyme methylguanine-DNA methyltransferase (MGMT) [39,40]. Therefore, high expression of MGMT counteracts TMZ-induced cytotoxicity and is also linked to a bad prognosis of patient survival [39,44]. Interestingly, the promoter of MGMT is methylated, i.e., no or reduced levels of the protein are produced, in 30 to 60% of GB patients [29,44,45]. Silencing of a tumor suppressor gene such as MGMT might occur during tumor development, thereby creating a more genetically unstable cell and favoring DNA damage and mutations, which ultimately would lead to better adapted tumor cell clones [44]. Therefore, one must consider the methylation status of the MGMT promoter as a prognostic marker for TMZ sensitivity [4,44]. The median overall survival of patients receiving TMZ-based chemotherapy and radiotherapy with a silenced MGMT promoter was 21.7 months compared to 12.7 months with active or, rather, an unmethylated MGMT promoter [44]. However, patients with a silenced MGMT promoter have a better clinical outcome irrespective of TMZ addition to radiotherapy and maximal safe surgical resection compared to patients with active MGMT [29,44,45,46].

**Figure 2:**
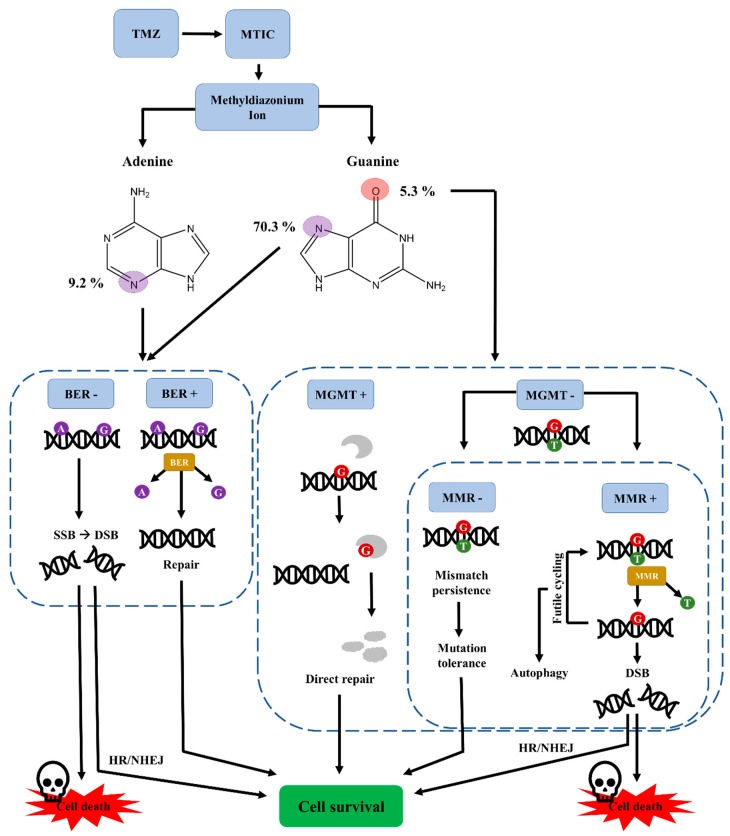
TMZ-induced alterations and DNA repair mechanisms involved in cellular response. The methyldiazonium ion, a highly electrophilic ion, methylates nucleophilic sites in the DNA. Lack of BER or low MGMT levels in combination with functional MMR are required for cell death induction. Abbreviations: BER, base excision repair; DSB, double strand break; HR, homologous recombination; MGMT, methylguanine-DNA methyltransferase; MMR, mismatch repair; MTIC, 5-(3-methyltriazen-1-yl)-imidazole-4-carboxamide; NHEJ, non-homologous end joining; SSB, single strand break; TMZ, temozolomide. Based on [29,40,50].

N7-methylguanine, which is prone to spontaneous depurination that forms toxic and mutagenic sites, and N3-methyladenine, which is per se highly toxic by blocking DNA polymerase, are easily repaired via BER [8,9,47,48]. Therefore, it is assumed that these lesions contribute much less to the cytotoxic effects. In contrast, O^6^-methylguanine, representing only a small fraction of all methylations, is considered to be particularly genotoxic and cytotoxic when mismatch repair is functional [28,49]. Figure 2 summarizes the proposed effects TMZ has on the cellular DNA.

## 4. Additional Functions of TMZ to be Considered

While research has almost exclusively focused on the effects and the consequences of genomic DNA damage by alkylation, it is important to keep in mind that the agents under discussion also have the potential to alkylate other macromolecules such as mitochondrial DNA, RNA, as well as proteins and lipids carrying nucleophilic groups [8,9,51,52]. Methylation of macromolecules is a post-transcriptional/translational modification, which is an important regulator of many different cellular processes. For example, mRNA folding and structure is altered, mRNA maturation is affected, nuclear processing and export out of the nucleus are enhanced, mRNA translation is promoted, and mRNA is marked for decay [53,54]. Methylation of proteins can affect chromatin structure remodeling, gene expression, DNA replication, synthesis and repair, the cell cycle, and apoptosis [55]. A classic example for the regulatory role of protein methylations are histone methylations, which define chromatin accessibility and thereby gene expression (reviewed in [56]). 

Protein methylation had been already discovered in 1959 [57], however, due to the limited knowledge and the lack of technologies at this time, it took almost half a century until this field of research evolved dramatically and the first pieces of evidence about the biological function of protein methylations were gathered [53,58]. Protein methylation is physiologically found at the side chains of at least nine amino acid residues [59], while it most commonly occurs at side chain nitrogen atoms of lysine and arginine residues, which influence protein structure, activity, localization, and interactions with other proteins [55]. 

Methylation of lysine and arginine residues in many proteins that are regulated via phosphorylation plays an important role in signaling pathways, such as mitogen-activated protein kinase (MAPK)-signaling, Janus kinase (JAK)-signal transducerand activator of transcription (STAT) signaling cascade, Wnt, and Hippo signaling [55,60]. The crosstalk between the two post-translational modifications (methylation and phosphorylation) allows definition of the strength and the duration of the signaling [61,62].

Although methylation of macromolecules is obviously a modification of great importance, it is rarely investigated and even ignored when discussing TMZ-mediated anti-tumor effects. In general, little has been done on that topic, and we found only a few reports showing primary data on macromolecule alkylation upon TMZ treatment. Experiments performed by Bull and Tisdale as well as experiments carried out by Wang and colleagues have shown that TMZ has the ability to methylate macromolecules in a cell-free system; TMZ is almost three times as effective in methylating calf liver RNA in comparison to calf thymus DNA [52], while TMZ also methylates bovine serum albumin and histone 3 recombinant protein [52,63]. However, little has been done in cell culture or in vivo. Bull and Tisdale showed that TMZ alkylates RNA and proteins after treating lymphoma cells, although fewer alkyl groups are bound to RNA compared to the cell free system. Interestingly, when treating human pancreas explants with *N*-methyl-*N*-nitrosourea (MNU), a monofunctional S_N_1-type methylating agent (and therefore of similar function as TMZ), the methylation of RNA was almost 12-fold higher compared to the methylation of DNA, while adducts were also found in the protein fraction [51].

Alkylating agents are also found endogenously and in our environment. Endogenous alkylating agents are, for example, bile acids or the methyl group donor S-adenosylmethionine, which is involved in many biochemical reactions. N-nitroso compounds found in tobacco smoke or food can alkylate the DNA as well [8]. Thus, it is not surprising that repair mechanisms for alkylation damage in RNA have been discovered [64,65], highlighting the importance of post-translational/transcriptional modifications such as methylations. Therefore, the methylation of macromolecules may play a much greater role in the mode of action of TMZ than it is currently accepted.

## 5. Open Questions Regarding TMZ’s Mode of Action

For many claims regarding the function of TMZ, data from other triazene compounds were extrapolated to TMZ without experimental verification. We further elucidate this problem by addressing two key questions that, in our opinion, have remained unresolved, although they are essential for future therapeutic optimization.

### 5.1. Membrane Permeability of MTIC

In aqueous solutions such as the blood, TMZ is spontaneously hydrolyzed to MTIC, which exerts the antitumor activity by breaking down into 5-aminoimidazole-4-carboxamide (AIC) and the methyldiazonium cation that subsequently alkylates the DNA [32,66]. TMZ is a rather small and lipophilic molecule (molecular weight 194 g/mol) leading to a rapid absorption, good tissue distribution, and BBB penetration [67,68,69]. Studies in adult male rhesus monkeys have shown that approximately 30–40% of TMZ plasma concentrations can be detected in the cerebrospinal fluids [70], while positron emission tomography studies using ^11^C-labeled TMZ allowed for demonstration of its neuropharmacokinetics in patients [71,72].

In contrast, MTIC, the active metabolite of TMZ, is reported to be unable to cross the BBB and cell membranes in general [30,34,73]. Additionally, the previously mentioned poor clinical performance of DTIC is sometimes attributed to the poor tissue distribution of MTIC [68]. In in vivo studies including mice bearing TLX5 lymphoma, MTIC could not be detected in the tumor [74]. As MTIC is a short lived metabolite in aqueous solutions (t_1/2_ ~ 2 min) [32], its detection requires fast tissue processing and highly sensitive assays, such as high performance liquid chromatography or liquid chromatography/mass spectrometry(MS)/MS, which were first validated in the late 1990s to detect MTIC in plasma samples [75,76,77]. Thus, it is possible that the lack of detectability in the tumor tissue was primarily due to technical limitations rather than poor tissue distribution properties of MTIC. 

Nevertheless, in vivo studies carried out in three Fischer 344 rats receiving a single intraperitoneal injection (i.p.) dose of [^14^C-methyl]-DTIC showed that, although DTIC was able to methylate the DNA in various tissues such as the liver, the kidney, and the lung, almost no methylation could be detected in the brain [78]. Meer and colleagues therefore hypothesized that the metabolites of DTIC, 5,3-hydroxy,ethyl-3-methyl-triazene-imidazole-carboxamide (HMTIC) and MTIC, possess a poor BBB penetration. Their report formed the basis for other papers claiming that MTIC is unable to penetrate cell membranes. Meer and colleagues, however, grounded their hypothesis on reports from Farquhar and Benvenuto [79]. Again, these reports contain only hypotheses based on observations, whereof two examples are mentioned here. First, in melanoma patients receiving DTIC, the tumor most frequently relapsed in the CNS, while the peripheral tumor could be controlled. Second, when testing carcinogenicity of DTIC in rats by chronic oral administration, tumors were observed mainly in the peripheral system and not in the CNS [79]. They claim that DTIC is unable to penetrate the BBB efficiently due to its low lipophilicity. However, studies from Bael and colleagues showed that ependymoblastomas and cerebral ependymomas could be induced after i.p. injection of DTIC into Sprague-Dawley rats [80]. In addition, radioactivity could also be detected in the brain after an i.p. injection of [^14^C-methyl]-DTIC.

On the basis of these reports, it is insufficient to conclude that the observations of Meer and colleagues are solely based on the fact that MTIC cannot cross cell membranes or the BBB without any independent experimental confirmation and further investigation addressing this aspect. Particularly because, at that time, the exact metabolism of DTIC and the tissue in which it takes place were still under investigation.

Unlike TMZ, which is directly hydrolyzed to MTIC, a chemically controlled reaction [33], DTIC requires oxidative N-demethylation via CYP450 enzymes and is converted into HMTIC, which subsequently eliminates formaldehyde and forms MTIC [22]. In rats, DTIC is N-demethylated by CYP1A enzymes [81,82], as was observed for humans as well [22]. In humans, CYP1A2 is primarily expressed in extrahepatic tissues including the brain, although the expression levels of CYPs in the brain are generally much lower compared to the liver. Consequently, it is thus far not known whether brain CYPs contribute to drug metabolism or not [83]. Therefore, there is no conclusive piece of evidence linking the inability of MTIC to penetrate the BBB to low frequency of methylations in the rat brain samples. To the best of our knowledge, we could find neither independent experimental confirmation of the experiments mentioned above nor further investigation addressing this aspect. As the underlying studies of Meer and colleagues included only three rats [78], validation in a larger set of samples is recommended. 

In addition, to the best of our knowledge, we could not find any primary data that would support the statement that MTIC is not able to penetrate cell membranes effectively. Several in vitro studies have shown that MTIC treatment of a variety of tumor cells (HeLa cells, colon carcinoma or lung adenocarcinoma cells, murine lymphoma cells) reduced cell viability as efficiently as DTIC and TMZ and induced DNA double strand breaks [84,85,86]. In those experiments, MTIC was simply added to the cell culture, thus it is likely that it is able to cross cell membranes in order to induce those biological effects. Notwithstanding, in most of the experiments, MTIC was dissolved in dimethyl sulphoxide (DMSO). DMSO is an amphipathic molecule and a commonly used organic solvent for lipophilic compounds tested in in vitro and in vivo experiments [87,88]. It easily penetrates cell membranes and mitochondrial membranes and seems to act as a carrier, enhancing the penetration of some compounds across membranes [87]. Therefore, it is possible that the effects seen by MTIC treatment in cell culture could be achieved by DMSO. However, Beal and colleagues could show that the growth of Novikoff hepatoma cells was inhibited by adding MTIC as a solid to the cell culture medium [89]. In addition, Sprague-Dawley rats that received MTIC, which was dissolved in 0.85% NaCl and 0.4% sodium carboxymethylcellulose and administered orally or by i.p. injection, developed a variety of tumors such as adenocarcinomas or leiomyosarcomas [80]. Therefore, they disprove the claim that MTIC cannot penetrate cell membranes and that MTIC effectiveness in other experiments is achieved because of DMSO salvation of MTIC. Nevertheless, independent experimental confirmation of these findings is necessary. 

Penetration of cell membranes does not imply that a substance also penetrates the BBB. Aiming to protect the brain and to maintain the special microenvironment, the BBB is composed of tightly packed endothelial cells, astrocytes, smooth muscle cells, and pericytes. Endothelial cells form tight junctions and thereby prevent paracellular diffusion and penetration of macromolecules into the brain. Additionally, endothelial cells express efflux pumps, which remove potential neurotoxic endogenous or xenobiotic molecules [90]. To address if MTIC has the potential to cross the BBB, we used admetSAR, an online tool developed to predict ADMET (absorption, distribution, metabolism, excretion, and toxicity) properties, including BBB penetration of molecules [91]. While it was only an in silico analysis, albeit with great predictive power, according to admetSAR, MTIC is almost as likely as TMZ to pass the BBB (probability: 0.9838 MTIC, 0.9925 TMZ).

### 5.2. DNA Targets of TMZ-Mediated Methylation

60–80% of the DNA adducts detected upon TMZ treatment are formed at the N7 position of guanine, especially in guanine rich sequences, and about 10–20% are formed at the N3 position of adenine. Only 5–8% of total DNA methylation occur at the O^6^ position of guanine [18,32,33,39,40,68]. This often-quoted assessment can be traced back to the PhD thesis of V. L. Bull [50]. In a cell-free in vitro experiment, he showed that TMZ was able to alkylate 16.49% of calf thymus DNA, while 70% of all adducts were associated with N7 guanine, 9.2% with N3 adenine, and only 5.3% with O^6^ guanine. 

In general, alkylating agents can bind to a variety of sites on DNA molecules (summarized in [9,92]), and many complex chemical models try to explain the substitution reaction of alkylating agents, i.e., the differences in the proportions of adducts formed at oxygen and nitrogen atoms in DNA [11,13]. However, when keeping it simple, the reaction basically follows the rules of electrophilicity and nucleophilicity [92]. The electrophilic alkylating agent is attracted to nucleophilic sites within the DNA, thus the distribution of DNA-alkylation adducts is heavily influenced by steric accessibility and electrostatic potential of the DNA. The N7 guanine, for instance, has the greatest negative electrostatic potential (−683 kcal·mol^−1^ [12]), which is why, in runs of guanines, the most electron-rich micro-environment is generated [32]. Furthermore, guanine triplet-rich sequences alter the DNA structure, leading to a wider major groove and greater steric accessibility [68] and facilitating the high rate of adduct formation at N7 guanine. In adenine-thymine base pairs, the minor groove including N3 adenine possesses the greatest negative electrostatic potential, which is why alkylation events occur there frequently [12]. Besides alkylating different positions within the bases, alkylating agents additionally form adducts at the ribose-phosphodiester backbone [7,92]. Furthermore, the alkylation pattern depends on the structure of the DNA, thus whether the DNA is single stranded or double stranded defines where hydrogen bonds will sterically hinder some nucleophilic sites [92]. The latter point is, however, contentious, as for other alkylating agents such as MNU, the DNA structure does not seem to have a great influence [8]. 

Furthermore, experiments support the hypothesis that alkylating agents do not randomly alkylate DNA but induce alkylation at specific genomic sites [93,94]. *N*-methyl-*N*′-nitro-nitrosoguanidine (MNNG), for instance, preferentially alkylates guanine-cytosine- and adenine-thymine-rich satellite DNA and repetitive sequences [94]. As those have structural regulatory functions, these alterations may be of great importance for the mode of action as well [94]. 

Taken together, the distribution of DNA-alkylation adducts appears to be an important factor for the biological effect. Consequently, it is fundamental to check for reproducibility of TMZ methylation patterns. Comparing data from cell-free in vitro experiments, the alkylation pattern of TMZ is quite similar to the ones of MNU and MNNG—both monofunctional S_N_1-type alkylating agents (see Table 1)—thus it could be possible that the three substances share a similar adduct distribution profile. Importantly, in vivo experiments utilizing MNU and MNNG show the same trend as in vitro experiments (summarized in [92]). Nevertheless, although considering steric accessibility and electrostatic potentials help to explain the preferences of adduct formation observed with different alkylating agents, the type of agent still influences the distribution pattern [7,8]. Therefore, it is fundamental to test if a similar distribution of methylations induced by TMZ can be observed in vivo, where synergistic or antagonistic interactions are involved. This would greatly help to understand the mechanism of action of TMZ and to find ways to support the effectiveness of TMZ in the clinic.

## 6. Conclusions

In this work we followed the historical development of alkylating agents for clinical use and highlighted the limits of our understanding of how the triazene family works on a molecular level. Furthermore, we identified essential questions that need answering when combining TMZ with novel therapeutic approaches to treat GB, essentially still an incurable disease, in the most effective way. 

GB is the most common primary brain tumor in adults, with an average age-adjusted incidence rate of 3.2 per 100,000 population [95]. It is also among the most lethal tumors per se, with only 5.5% of patients surviving five years or more after diagnosis [96]. As GB is much rarer in children and adolescents, it is often overlooked in a pediatric context, although overall survival is only marginally better than in adults [97].

The growth pattern of GB is diffuse and highly invasive, and upon clinical presentation, the surrounding brain tissue is invariably infiltrated [2]. Even after maximal safe surgical resection of the tumor bulk, the growth recurs within 2–3 cm of the resection cavity in 95% of all cases [2]. Therefore, localized treatment such as surgery and focal radiotherapy must be considered particularly ineffective [98], and the whole brain should be considered a therapeutic target, e.g., GB is a systemic brain disease.

Unusual for such an aggressive tumor, GB exhibits a low to moderate mutational burden [99,100], i.e., it presents few tumor-specific druggable targets, and no driver mutations have been identified. The most common alterations are found in the PI3K pathway, which is activated in almost 90% of all glioblastoma [99,101,102]. However, while this signaling network is often considered to mediate survival, its role in GB cell subpopulations is more complex [103,104], and its modulation has thus far not translated into clinical success [105,106]. Another common alteration in GB is the promoter methylation of the MGMT gene, which occurs in 30–60% of all GB patients, although with certain heterogeneity within the tumor [29,44,45]. It is the low or the absent expression of this DNA repair enzyme in GB cells that makes TMZ the backbone of GB therapy, being the only systemic compound of the standard therapy.

The therapeutic efficacy of TMZ is limited; early clinical data clearly indicate that only a small percentage of patients experience a substantial reprieve, and median survival was only extended by 2.5 months [3,4]. It is worth remembering that MGMT is a suicide enzyme, i.e., one TMZ-induced lesion is repaired by one MGMT molecule that is destroyed in the process [39,40]; therefore, a theoretic strategy could be envisioned whereby MGMT is depleted from the tumor by metronomic treatment with an alkylating agent. However, alkylating agents are toxic, and the often (over)stated comparatively mild side effects of TMZ in contrast to potent chemotherapeutic agents such as doxorubicin, for example, were selected by sacrificing drug potency [17,21]. From the beginning, TMZ was a compromise because we had nothing better to offer, which might also explain the lack of research into its mode of action.

However, now that TMZ has become the standard and—this is worth repeating—does help some patients greatly, it will be a significant component in any future clinical trials (as part of the control group as well as the novel treatment group), and it also has a place as part of complex combination therapies, such as RIST (rapamycin, irinotecan, sunitinib, temozolomide) [107] or CUSP9 (therapeutic regime which includes nine repurposed drugs along with low-dose TMZ) [108]. Therefore, understanding what TMZ does on a cellular level has important implications for combination therapies, where several substances and several classes of substances (small molecule inhibitors, antibodies, oncolytic viruses, as well as classic chemotherapy) are combined in a strict temporal sequence. One could easily envision a combination where the effect of TMZ hinders the potency of the combination. For example, the application of alternating electric fields (TTFs) has recently gained FDA approval after a clinical trial demonstrated an increase in median overall patient survival (20.9 months versus 16.0 months) [109]. TTFs are proposed to interfere with cell division, in essence preventing the chromosomes from equatorially lining up during mitosis [109]. Indeed, it has long been proposed that chemotherapeutic reagents exert their highest potency in cycling cells [110], and interfering with mitosis is a promising therapeutic strategy [111]. In contrast, induction of quiescence is believed to be chemoprotective [112], and the slow-cycling nature of potential cancer stem cells is believed to contribute to their therapy resistance [113]. Thus far, the only consistently shown effect of TMZ on cells is the increase of DNA content. Whether this is due to a G2 arrest or under-replicated DNA [114] remains to be experimentally validated. While TMZ has been shown to induce cell death, this is usually only produced in experimental systems with un-physiologically high concentrations, often in the range of 100 µM TMZ [41,42,115,116,117] up to 1000 and 4000 µM [118,119,120,121], while models predict a peak concentration in the tumor in the range of 14.95–34.54 µM [122]. Possibly, TMZ should be considered primarily cytostatic and senescence-inducing and not cytotoxic and apoptosis-inducing [123], potentially preventing cancer cells from G2 to M phase transition when tumor cells are most sensitive for mitotic cell death.

In summary, the molecular modes of action of alkylating agents such as TMZ are still not fully understood, and the differences between individual members of the triazene family are often downplayed. As TMZ is currently the only systemic component of GB standard therapy, it is essential. However, by not understanding its mode of action fully, we risk that TMZ might mask the potency of novel therapeutic approaches.

## Figures and Tables

**Table 1:** Distribution of alkylated adducts in the DNA upon treatment with monofunctional S_N_1-type alkylating agents. Methylation of DNA is expressed as percentage of total alkylation upon stimulation with temozolomide (TMZ), *N*-methyl-*N*-nitrosourea (MNU), or *N*-methyl-*N*′-nitro-nitrosoguanidine (MNNG). (a) Data from [50]. (b) Data from [92]. Dash: data not reported; nd: adduct not detected or below detection limit of the respective assays used.

	In Vitro Methylation Pattern (Cell-Free System)			Adduct Profile of Cultured Cells	Adduct Profile of Bacteria	Adduct Profile of Isolated Rat-Liver DNA	
	Percentage of total alkylation/ binding to DNA [%]						
**Site of Alkylation**	TMZ ^a^	MNU ^b^	MNNG ^b^	MNU ^b^	MNNG ^b^	MNU ^b^	MNNG ^b^
**Adenine**							
**N1**	-	0.7–1.3	1.0	nd	-	0.3	-
**N3**	-	8.0–9.0	12.0	3.8–4.2	2.0	1.1–3.6	8.6
**N^6^**	-	nd	-	-	-	-	-
**N7**	9.2	0.8–2.0	-	1.5–3.1	-	0.8	-
**Cytosine**							
**O^2^**	-	0.1	-	nd	-	nd	-
**N3**	-	0.06–0.6	2.0	0.3–0.4	-	nd	-
**Guanine**							
**N1**	-	nd	-	-	-	-	-
**N3**	-	0.6–1.9	-	0.5–0.7	-	0.6–1.6	-
**O^6^**	5.3	5.9–8.2	7.0	5.1–11.6	11.0	3.6–10.0	9.2
**N7**	70.3	65.0–70.0	67.0	69.0–72.9	78.0	70.0–86.6	82.2
**Thymidine**							
**O^2^**	-	0.1–0.3	-	nd	-	nd	-
**N3**	-	0.1–0.3	-	nd	-	nd	-
**O^4^**	-	0.1–0.3	-	0.5	-	1.8	-
**Phospho-triesters**	-	12.0–17.0	-	9.0–11.7	-	13.4	-
